# Unexpected Radiation-Induced Aortic Wall Thickening Requiring Composite Graft Technique during Off-Pump Coronary Artery Bypass Grafting

**DOI:** 10.1155/2017/3831749

**Published:** 2017-01-19

**Authors:** Paola Redaelli, Amando Gamba, Antonello Stefano Martino, Michele Triggiani

**Affiliations:** Cardiovascular Department, Cardiac Surgery Unit, “A. Manzoni” Hospital, Lecco, Italy

## Abstract

Mediastinal radiation is commonly used to treat Hodgkin's and non-Hodgkin's lymphoma, lung and breast cancer. Cardiac complications after radiation therapy are well described, although rare. A large spectrum of injuries can occur, causing long term morbidity among survivors. We describe a case of post-actinic ascending aortic wall thickening that prevented saphenous vein proximal anastomosis and was successfully managed with aortic no-touch off-pump coronary artery bypass grafting (OPCAB), 25 years after radiation therapy for Hodgkin's lymphoma.

## 1. Case Report

**Figure 1:**
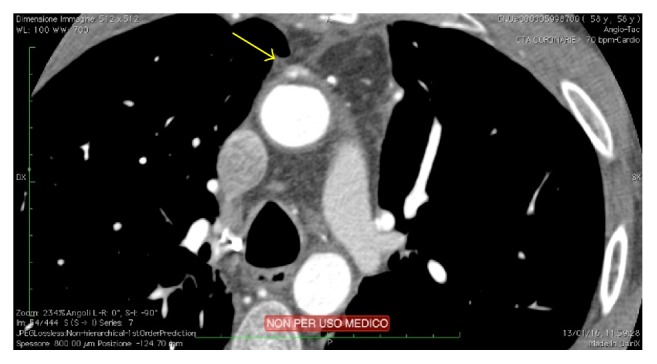
Thoracic CT scan of the patient two months after OPCAB. The yellow arrow points at a localized anterior wall disruption on ascending aorta, at the site of aortic punching.

**Figure 2:**
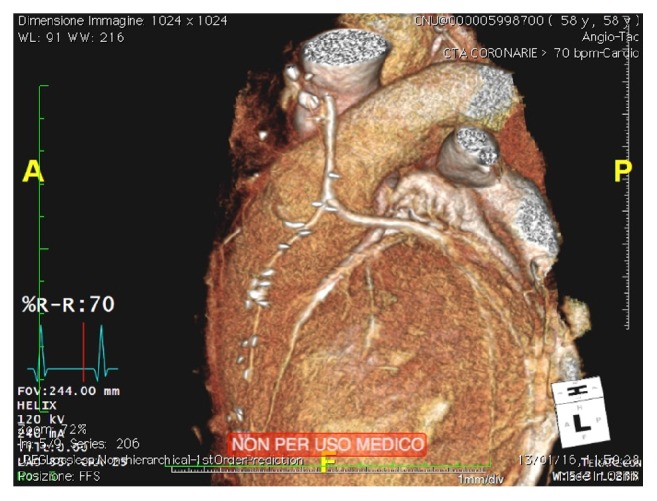
3D reconstruction of the coronary CT scan, showing a patent LITA to LAD and a composite Y-graft with SV from LITA to obtuse marginal branch.

A 58-year-old man was admitted to our hospital because of acute cardiac decompensation. A transthoracic echocardiogram showed increased left ventricular end-diastolic volume and diameter (160 mL and 55 mm, resp.), severely compromised left ventricular ejection fraction (LVEF 35%) with diffuse hypokinesia, and diastolic grade I dysfunction. A mild mitral regurgitation (MR) was noticed, while aortic valve leaflets and function were normal. Left cardiac catheterization showed 50% stenosis of left main and chronic total occlusion of the right coronary artery; therefore surgical myocardial revascularization was scheduled. The medical history of this patient was significant for impaired glucose tolerance, previous smoking, and Hodgkin's disease (HD) when he was 33 years old. Because of this, he was submitted to mediastinal radiation therapy (RT), with negative follow-up thereafter. In consideration of history and clinical presentation, we decided to perform OPCAB, harvesting left internal thoracic artery (LITA) and a segment of saphenous vein (SV). Despite the presence of firm adhesions at harvesting, LITA flow and caliber were proper, so that this conduit was successfully grafted to left anterior descending artery (LAD). Ascending aorta (AA) and pulmonary artery were partially covered by fibrotic tissue, and dissection to allow side clamping of the aorta was demanding. Finally, we did not succeed in punching the aortic wall, because it was abnormally hyperplastic; we decided to change strategy, preparing a composite Y-graft (SV to LITA) for obtuse marginal branch. During the operation it was not possible to achieve our standard target activated clotting time (ACT) for OPCAB (>250 sec), despite high doses of heparin and antithrombin III. The immediate postoperative course was characterized by an intense inflammatory response. On first postoperative day dual antiplatelet therapy with acetylsalicylic acid and clopidogrel (100 mg and 75 mg daily, resp.) was started. The patient moved to the Cardiologic Rehabilitation Ward on the third postoperative day, and postoperative course was uneventful. He was discharged 15 days after surgery. Two months later, the patient underwent thoracic and coronary computed tomography (CT) scan that confirmed severe thickness of the ascending aortic wall (5.6 mm versus 2 mm normal value) and a localized wall disruption at the site of aortic punching (Figure 1). All grafts were patent (Figure 2).

## 2. Discussion

Mediastinal RT can cause several cardiac injuries [1, 2]. In literature the incidence of coronary artery disease (CAD) after RT is 10%, with a latency of onset between 1 and 32 years, while cardiovascular morbidity is reported to be 16% at 20 years [3]. In our patient RT was likely responsible for hypokinetic dilated cardiomyopathy, impaired diastolic function, and CAD. Post-RT coronary lesions usually involve the ostium or the proximal part of left main and right coronary artery, probably because they are located anteriorly and exposed to a high radiation dose [1, 2]. The preferable conduits and the ideal technique to perform coronary artery bypass grafting (CABG) in post-RT patients are not well defined. In past years there has been a great debate over the use of an irradiated LITA: its patency rate was found to be similar to venous or radial arterial conduits, but patient series are too small to draw definitive conclusions, and results are conflicting [4, 5]. Considering that cardiac operations in patients with previous hematological malignancies are associated with higher rates of complications and mortality (up to 18.7%) [6], we planned OPCAB, expecting this technique to be beneficial. Right coronary artery was chronically occluded, and we chose to perform CABG only on LAD and obtuse marginal branch. After AA side clamping, an abnormally thick aortic wall prevented us to perform the proximal vein anastomosis, because an adequate aortic incision could not be achieved. We decided for a composite Y-graft between LITA and SV, and surgical strategy was easily changed thanks to the off-pump technique. Post-HD patients are also known to develop intraoperative or postoperative hypercoagulability/bleeding [6], and the commonly accepted lower ACT in OPCAB may be helpful. In conclusion, we suggest to perform a preoperative thoracic CT scan in patients undergoing surgical myocardial revascularization and previously exposed to mediastinal RT because of the risk of radiation-induced aortitis [2]. Moreover, OPCAB may be advantageous in post-RT patients because it allows multivessel revascularization avoiding any manipulation of the AA, potentially damaged by previous RT. LITA with a proper flow remains probably the conduit of choice for LAD, allowing composite Y-graft.
